# WiFi FTM, UWB and Cellular-Based Radio Fusion for Indoor Positioning

**DOI:** 10.3390/s21217020

**Published:** 2021-10-23

**Authors:** Carlos S. Álvarez-Merino, Hao Qiang Luo-Chen, Emil Jatib Khatib, Raquel Barco

**Affiliations:** Instituto Universitario de Investigación en Telecomunicación (TELMA), University of Málaga, CEI Andalucia TECH E.T.S.I. Ingeniería de Telecommunication, Bulevar Louis Pasteur 35, 29010 Málaga, Spain; hao@ic.uma.es (H.Q.L.-C.); emil@uma.es (E.J.K.); rbm@ic.uma.es (R.B.)

**Keywords:** indoor positioning, fusion technologies, UWB, WiFi fine time measurement, LTE, maximum likelihood estimator

## Abstract

High-precision indoor localisation is becoming a necessity with novel location-based services that are emerging around 5G. The deployment of high-precision indoor location technologies is usually costly due to the high density of reference points. In this work, we propose the opportunistic fusion of several different technologies, such as ultra-wide band (UWB) and WiFi fine-time measurement (FTM), in order to improve the performance of location. We also propose the use of fusion with cellular networks, such as LTE, to complement these technologies where the number of reference points is under-determined, increasing the availability of the location service. Maximum likelihood estimation (MLE) is presented to weight the different reference points to eliminate outliers, and several searching methods are presented and evaluated for the localisation algorithm. An experimental setup is used to validate the presented system, using UWB and WiFi FTM due to their incorporation in the latest flagship smartphones. It is shown that the use of multi-technology fusion in trilateration algorithm remarkably optimises the precise coverage area. In addition, it reduces the positioning error by over-determining the positioning problem. This technique reduces the costs of any network deployment oriented to location services, since a reduced number of reference points from each technology is required.

## 1. Introduction

Location-based services in the fifth generation (5G) mobile network require reliable, continuous, and precise positioning information for their full functionality potential [1]. Global navigation satellite systems (GNSS) have settled as the reference localisation system for outdoor navigation. GNSS offer a meter-level accuracy at open sky scenarios. However, the precision is reduced drastically when the target enters a building or tunnel. Several technologies (e.g., WiFi and Bluetooth) and techniques (e.g., fingerprinting and image recognition) try to provide accurate and precise location information [2,3]. Nevertheless, indoor scenarios are extremely challenging due to the harsh radio propagation conditions. Indoor scenarios usually contain metallic objects that reflect and block the signals creating multipath effects that can strongly deteriorate the navigation solution or create areas where no navigation information is available. Moreover, typical indoor scenarios are dynamic with constant changes due to the mobility of people within the scenario, such as in a shopping mall or an office. High-precision positioning becomes crucial for some Internet of Things (IoT) services, such as augmented reality (AR) or context-aware applications.

In recent years, some technologies have emerged for precise indoor localisation. There are two main families of techniques: based on trilateration, and based on fingerprinting. Trilateration consists in obtaining the position of the target based on the intersection of the distance between the target and at least three reference points. Some technologies that are being studied are ultra-wide band (UWB) [4], WiFi fine-time-measurement (FTM) [5] and cellular-based radio [1]. UWB has been widely adopted due to its robustness against multipath and its centimetre-level accuracy [6]. It may become the standard for indoor positioning in the next years as defined by ETSI [7]. Since UWB devices have a range of up to few tens of metres, they need a fairly dense deployment to ensure the required coverage. However, a dense deployment of UWB in the real world has a very high cost, making it feasible only for limited scenarios. WiFi FTM relies on the wide availability of WiFi access points (APs), and the higher range of WiFi signals to reduce the deployment costs, but it is still a fairly new technology that has not a wide commercial adoption. Although 5G ranging is still under research [8], it promises a very high availability thanks to the omnipresence of 5G base stations. Long term evolution (LTE) has also been used for obtaining location, although its precision is not as high as UWB or WiFi FTM [9].

WiFi fingerprinting [2,3] has also been widely studied. In fingerprinting, instead of using reference points, the terrain is divided in a lattice, and for each division, the set of visible WiFi APs is collected in an offline stage. For estimating location, a target will then obtain a list with the visible APs, and will use it to find the point in the lattice where it is most likely located. The map must be frequently updated to reflect changes in the environment.

In this work, we propose a method for opportunistically aggregating ranges obtained from different technologies. This fusion technique helps to reduce the cost of deployment because the end-user benefits from any nearby ranging reference point (RP) for localisation [10]. In addition, this technique also helps dealing with coverage holes of certain deployments, that is, areas where there are less than three visible reference points of one technology. Since not all ranges have the same precision, we propose a weighting stage that prioritises the reference points that offer a better location quality. To this end, this work uses a maximum likelihood estimator (MLE) to characterise the ranges and sources in order to define the weighting algorithm to balance the information of the over-determined system which provides high accuracy indoor positioning.

To validate the proposed method, we use a real location deployment with ranging information based on time measurement from UWB and WiFi and received signal strength from a LTE network as a back-up. To the best of our knowledge, no system unifies all these three technologies and brings them into a real scenario to show the performance of real-time localisation. We also test several search methods for the MLE, to compare the advantages in location precision and computational time of each one.

The contribution of this paper is listed as follows:

- Proposition of a fusion method in trilateration based on the work presented in [10], with a dynamic weighting with MLE that improves the robustness of location accuracy;
- Validation of the proposed method with a real-world setup with several different scenarios;
- Comparison between different MLE search methods for finding the best for resolving over-determined location problems.

The rest of this paper is organised as follows: Section 2 provides an overview of the different location technologies explaining features of the technologies used in this work. Section 3 explains the proposed method and the algorithm of multi-technology fusion and the MLE as a weighting technique for outliers. Section 4 describes the experimental setup with two scenarios and Section 5 presents the results obtained from three different cases deployed in the two scenarios. Section 6 discusses the results presented in the previous section. Finally, Section 7 presents the conclusions of this work.

The acronyms in this paper are listed in the Table 1 as follows:

## 2. Overview of Location Technologies

The focus of the cellular-user location has changed over the generations from outdoors to indoors [1]. Thus, GNSS has had to adapt to the new requirements. However, other technologies and techniques have overcome satellite-ranging solutions for indoor positioning. Ranging-based or fingerprinting location have been studied [11,12] to provide a high accuracy for indoors with technologies presented in Table 2, such as Bluetooth or WiFi. Some of these technologies have been discarded for this work for several reasons. First of all, the scope of this work is to study the real-time positioning with technologies that do not need the data collection phase in fingerprinting, such as geomagnetism [12]. Secondly, user location must be computed in the cloud for two main reasons: it will help future applications, such as driver-less cars in which the cloud runs the commands to the cars and must know their positions [13], and computing in the cloud also helps reducing energy consumption in the end-device [14]. Thus, inertial navigation system (INS) is excluded for this study because it is unfeasible to send the information of the accelerometer or gyroscope in real time (update rate ≥ 100 Hz) with high energy efficiency. Thirdly, low-stability technologies, such as Bluetooth, might not allow to update in real-time, for the RSSI variations [15]. Moreover, Bluetooth provides an insufficient coverage for wide scenarios [16]. Hence, we decided to discard Bluetooth for this indoor positioning study. Henceforth, the technologies that are finally studied in depth for indoor positioning with high-precision performance are UWB and WiFi FTM. LTE is also studied as a back-up technology due to the wide-area coverage and deployed infrastructure. Table 2 presents an overview of the technologies mentioned in this study.

Hereinafter, a brief description of cellular-based radio, UWB and WiFi that will be used in this paper is completed.

### 2.1. Cellular-Based Radio

Cellular-based localisation has been used as a simple and coarse solution when there is a lack of satellite visibility in GNSS, typically indoors and in scenarios, such as urban canyons [17]. The arrival of 5G brings new specifications for high-precision positioning as described in [9], which can be summarised in:

- Horizontal and vertical positioning error < 3 m for 80% of user equipments (UEs) in indoor deployments;
- Horizontal and vertical positioning error <10 m and <3 m, respectively, for 80% of UEs in outdoors deployments.

5G works on 700 MHz, 3.5 GHz and millimetre wave of 26 and 28 GHz. High frequencies allow high-precision ranging in direct line of sight (LoS) with the target but highly suffers from attenuation, multipath and reflections in non-line of sight (NLoS). In contrast, lower frequencies are more robust to attenuation reaching longer distances, however, multipath effects can deteriorate the precision of the ranges. In [1], in order to eliminate the need for clock synchronisation, the use of different timing techniques such round-trip time (RTT) are proposed for indoors.

Nevertheless, the existing and deployed LTE networks can be used as a back-up for other location technologies [10]. Despite of the coarse ranging information that LTE provides, LTE network is globally available in contrast with 5G that has not been yet fully deployed. End-users may benefit from LTE in cases where no high-precision technologies provide localisation. However, LTE utilises the received signal strength indicator (RSSI) for ranging. RSSI highly suffers from multipath and fadings which leads to high variations and an increase in the ranging error.

### 2.2. Ultra-Wide Band

UWB technology provides a high ranging accuracy based on the RTT protocol, even in environments with harsh propagation characteristics [18]. This technology has multiple advantages, such as centimetre-level ranging precision, good obstacle-penetration capabilities [4], and multipath mitigation in dense scenarios [2], making it indispensable for indoor positioning. UWB is also a wireless communication technology that supports a high throughput owing to the use of a very large spectrum. UWB uses very short time pulses of few nanoseconds that take a wide bandwidth. The Federal Communication Commission (FCC) authorised the unlicensed use of UWB in the range of 3.1 to 10.6 GHz [3]. UWB signals are centered at 3.5 GHz with a bandwidth higher than 500 MHz. The latest market trends show that UWB will soon become a de-facto standard for positioning and will eventually be addressed by 3GPP standards [7]. Accordingly, some smartphones have integrated UWB chipsets in the recent years [19]. As a drawback, to achieve the short pulse width the UWB device has a high energy consumption.

### 2.3. WiFi Fine Time Measurement

IEEE 802.11mc includes a fine time measurement (FTM) for range estimation in timing protocols using RTT [20,21]. This release will transform the indoor positioning industry in the next years because WiFi infrastructure is widely deployed. The protocol estimates precisely the distance to any WiFi access point (AP) which supports the protocol without needing to be connected to them [22]. The information is calculated on the device for privacy preserving, since sensitive location information is not shared among network peers. In [23], the accuracy for positioning of WiFi FTM is computed with a precision of a meter-level accuracy in real scenarios with dense deployments of WiFi APs.

## 3. Materials and Methods

### 3.1. Proposed Positioning Method

In trilateration, the position of the target is in the intersection between geometric forms, such as circles or hyperbolas defined by the distance between the target and the RP [1,2,3]. Any ranging information can be used to obtain the final target position, such as time of arrival (ToA), RSSI, or RTT time measurements. A minimum of three sets of reference points and ranges to each one is required for location in 2D. The proposed algorithm is explained in Algorithm 1. First, once the ranging information is received, the MLE weights each source depending on whether the source is new or the system already has information about it. Then, the trilateration algorithm based on the weighted least-square (WLS) algorithm is computed [24]. Once the position is obtained, the algorithm computes the error based on the distance provided by the source and the computed position. Finally, this error is temporarily stored and the weighting factor of the source is updated for the next iteration. In this section, both techniques that will be used in this paper are described: multi-technology fusion and maximum likelihood estimation (MLE).
**Algorithm 1:** Positioning algorithm with MLE and fusing technologies
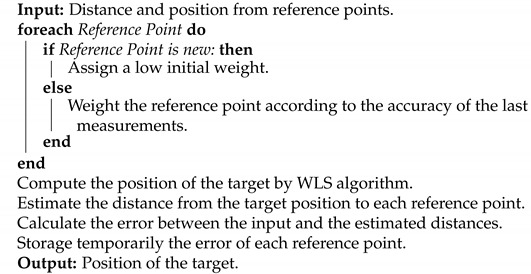


**Figure 1:**
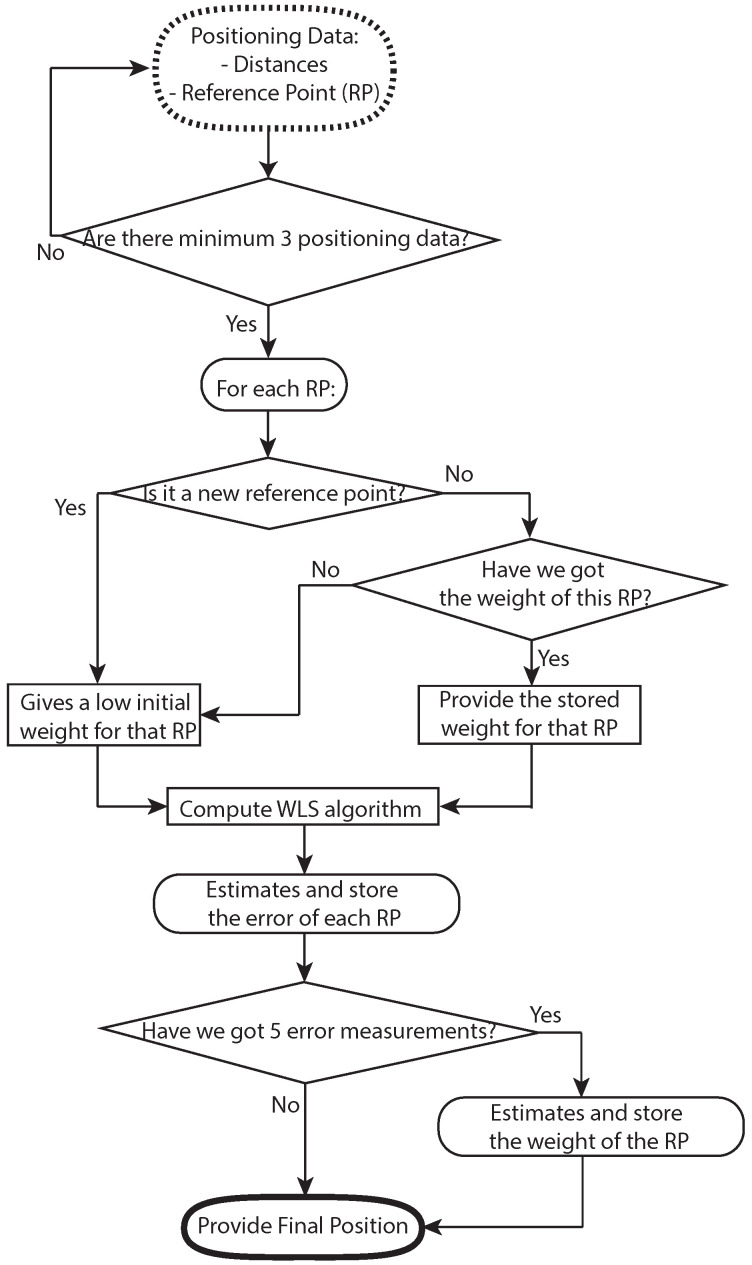
Flowchart of the system.

For a better understanding, we have also provided a flowchart of the proposed positioning method that will help to understand how the whole system works in Figure 1.

### 3.2. Multi-Technology Fusion

In trilateration the ranging information usually comes from a single technology. However, in [10], a scheme for fusing ranges from different technologies is presented. The use of multi-technology fusion in trilateration improves low-precision accuracy provided by technologies (in this work, LTE) by using the ranges of precise technologies, such as UWB or WiFi FTM.

In addition, to enhance the end-user location precision, multi-technology fusion also provides a seamless navigation between areas served by different technologies (e.g., exteriors where GNSS can be used, and interiors with UWB deployments, using other ranging technologies, such as LTE to cover for the missing ranges in the borders). Fusion benefits from high-precision reference points in a sparse deployment which can help to improve location in emergency cases, such as fires, earthquakes, etc. In these scenarios, fusion can also compensate the missing AP structures with portable APs in order to provide high-precision localisation. Although in this paper we have set the focus on WiFi FTM and UWB for precise positioning, other technologies which provide high-precision ranging data could also be used, such as GNSS or Bluetooth.

### 3.3. Maximum Likelihood Estimator (MLE)

Ranging information is uncertain because measurements are never ideal. Least squares (LS) estimation solves over-determined systems even though they are inconsistent since it is not possible to find a solution. The idea is to find a solution which minimises the error of the system. However, the classical iterative LS method for positioning lacks robustness; even a single outlier can introduce a great error in the estimated target position. This problem increases when the final accuracy should be reduced to the sub-meter accuracy. In [10], WLS was used to compute the location with trilateration, but to avoid the problem of outliers, the ranges were weighted according to their precision. A higher weight was assigned to UWB (which is more precise) than LTE. However, these weights were assigned statically; so, if a specific device introduced a high-precision ranging error (due to factors such as an especially challenging location for propagation, or software and hardware malfunctions), the localisation accuracy would be considerably affected.

Maximum likelihood estimator (MLE) is the most popular estimator for obtaining the parameter $\hat{\theta }$, which specifies a probability function P(X=x|θ) or a probability density function p(X=x|θ) of a discrete or a continuous variable based on the observations $x_{1},x_{2};\ldots ,x_{n}$ which are independently sampled from the distribution [25]. In this work, MLE weights the ranges provided by different reference points in real-time depending on the variation of the error attached to the ranges. The system stores the error associated to each RP iteratively with a temporal window and weights the sources by their standard deviation. Supposing that $X=\{X_{1},X_{2},\ldots ,X_{n}\}$ with distribution $F_{\theta }\;\mathrm{being}\;\theta =\left\{\theta_{1},\theta_{2},\ldots ,\theta_{n}\right\}$ that follows the density function $f_{\theta }\left(x\right)$ [26]. Hence, the likelihood function of the observation is given by:

(1) $$L\left(\theta ;X\right)=\prod_{i=1}^{n}f_{\theta }\left(X_{i}\right)$$

The MLE estimates the best candidate that optimally maximises *L* as seen below:

(2) $$\hat{\theta }=argmax\left(log\left(L(\theta ;X)\right)\right)$$

**Figure 2:**
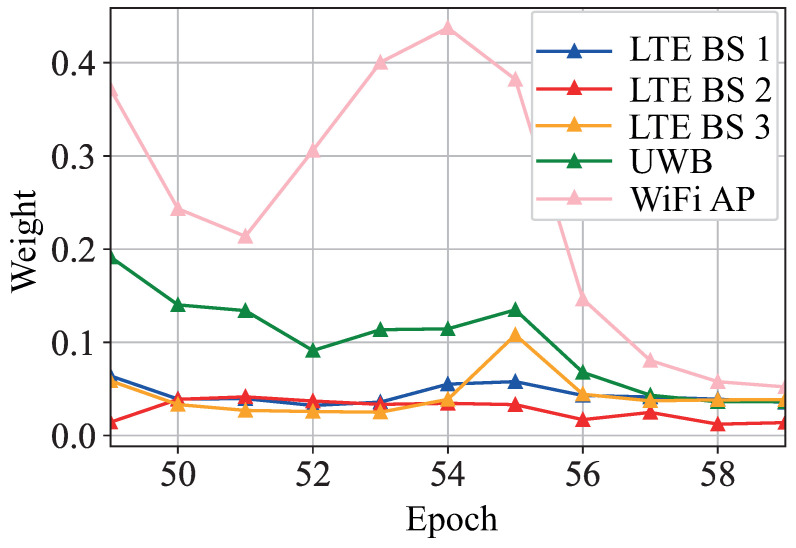
Functionality of the MLE during a real experiment.

Hence, assuming that observations follow a Gaussian distribution, the estimator calculates the parameters of mean and standard deviation that best suits Equation (2). In this work, MLE dynamically weights the different reference points at the WLS algorithm. Once the target’s position is estimated, the distances (${\hat{d}}_{\mathrm{est}}=\left\{{d_{est}}_{1},{d_{est}}_{2},\ldots ,{d_{est}}_{n}\right\}$) from the estimated position to each RP positions are calculated. Then, the estimated error of each RP (${\hat{e}}_{\mathrm{est}}=\left\{{e_{est}}_{1},{e_{est}}_{2},\ldots ,{e_{est}}_{n}\right\}$) between the estimated distances (${\hat{d}}_{\mathrm{est}}$) and the input distances (initially for the LS algorithm) are obtained. Finally, MLE provides the weight values of each RP based on the error (${\hat{e}}_{\mathrm{est}}$) from the last *N* time epochs. The value of *N* depends on the periodicity that the measurements are captured. We store the *N* elements that were captured in the last 5 s. Figure 2 represents the weighted values of different reference points during an experiment. In this case, it can be observed that a WiFi AP is overweighted for some epochs. Then, owing to a blocking of line of sight between the target and the WiFi AP, the weight of the WiFi AP is reduced drastically due to the precision of the range being reduced to the level of lower precision technologies such as LTE base stations (BSs).

Thus, the system benefits from the most stable and precise ranges. When the MLE receives a new input source (i.e., a new RP and its distance), the estimator assigns a low weight during the first *N* epochs in order to check the stability of the new source. In case a RP data are not captured, the MLE erases the stored data of that RP. Once all the information is weighted, WLS algorithm utilises the weights to provide the best target position. To find the solution, several searching techniques can be used for the MLE:

- Nelder–Mead: is the most widely used algorithm in direct search method for solving the unconstrained optimisation problem. The Nelder–Mead method iteratively generates a sequence of tetrahedrons to approach the optimal point which can reflect, expand, contract, and shrink. The algorithm is designed for small search spaces because it quickly stalls [27];
- Limited-memory Broyden–Fletcher–Goldfarb–Shanno (L-BFGS): is designed for large non-linear optimisation problems. The algorithm handles bounds on the variables and solves unconstrained problems. However, the convergence is slow and non-optimal for real time cases [28];
- Truncated Newton (TNC): utilises rougher estimations of the optimal search direction for efficiency. As a drawback, the algorithm appears to rapidly stall [29];
- Constrained optimisation by linear approximation (COBYLA): is a direct search method which only incorporates linear models about the objective and the constrains with quick searching time [30];
- Sequential least squares programming (SLSQP): is an iterative method in which the objective and constraints functions demand to be triple continuously differentiable. The method reduces the non-linear optimisation problems by sequential iterations to trim the convergence time [31].

In this paper, the SciPy [32] package implementation of these algorithms was used.

## 4. Experimental Setup

In this section, an experimental setup for validating the solution is described. To validate the benefits of the proposed solution with real data, an UWB and a WiFi FTM deployment are used as high-precision ranging technologies and an indoors LTE network as a backup element with low-location accuracy and high availability. LTE is used as a placeholder of 5G due to the lack of an experimental infrastructure, but the conclusions of the experiment are expected to be similar with femtocells in a height of 3.5 m. The LTE network belongs to the University of Malaga which has configured the network to reduce the interferences with commercial networks. On the other hand, according to the multipath effects, the scenario is a laboratory which presents several metallic elements, such as computers, shelves, etc. Therefore, we expect that the measurements are heavily affected by multipath. The UWB deployment is based on Decawave DWM1000 devices (DecaWave, Dublin, Ireland) and they were placed on top of shelves in order to cover the whole scenario with good visibility (2 m height). Meanwhile, the WiFi FTM APs are Google WiFi mesh routers (Google, Montain View, CA, USA) that were placed in typical places for providing WiFi connectivity throughout the laboratories (1 m height). Both DWM1001 and Google WiFi routers are set to their default configuration parameters [33,34]. The UWB devices transmit with a power of −14.3 dBm and they are centered in 6 GHz [34]. The Google WiFi routers are configured to work at 2.4 GHz and, to the best of our knowledge, the WiFi RTT FTM function could not operate at 5 GHz. The transmission power of the router is by default 28.17 dBm [33]. The LTE station parameters are configured with a transmission power of −6.8 dBm and downlink and uplink frequencies of 2630 MHz and 2510 MHz, respectively. The location target device is a Google Pixel 3 which runs Android 9.0 and supports WiFi FTM RTT. An application has been programmed to capture all the ranging data from the network reference points: LTE base stations, UWB anchors and WiFi APs. The ranges with the LTE stations are estimated using the measured RSSI which is modelled by the indoor office propagation model [35]. The WiFi FTM ranges are obtained from an API created for this work. For the UWB measurements, a DWM1000 device is attached to the smartphone and connected via Bluetooth low energy (BLE) to read the UWB data. A limitation on the performance of the UWB devices is that the UWB tag can only receive the information of four anchors simultaneously due to the software provided with the DWM1000 family products [4]. The implementation of the positioning and networking stack (PANS) firmware, the two-way ranging (TWR) communication protocol and the data frame limit the number of anchors that the tag can listen at the same time. Hence, despite a high-density set-up, the system is not highly over-determined. The captured data are sent to a Flask server with a MySQL database which is configured in a laptop(Lenovo, Beijing, China) running Windows 10. The sampling rate is 1 Hz. The measurements are timestamped with the global satellite system (GPS) clock as a time reference. The measurements are captured in a reduced time interval, assuming simultaneous samples which would introduce some error due to synchronisation. However, a regular user moves slowly in indoor scenarios which can lead to a maximum error of a few centimetres of ranging information. Additionally, we have set the height of the phone to 1.1 m of height simulating a person is carrying the phone on his pocket. Then, we send the information to the Flask server to process the localisation data via HTTP with no retransmission allowed in order to maintain the experiments as real-time.

**Figure 3:**
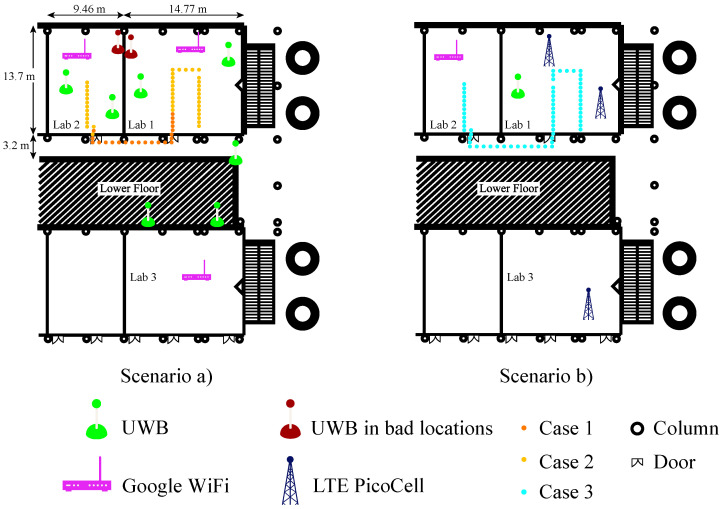
High density (**a**) and low density (**b**) scenario set-up distribution.

These experiments were performed in three laboratories and hallways with a very limited vision of the sky. Figure 3 illustrates the three experimental cases that have been measured in the two different scenarios. These cases were selected in order to demonstrate the advantages of fusing technologies and the performance of the MLE and its different searching methods for localisation. Two experimental cases were carried out as shown in the high-density deployed scenario illustrated in Figure 3a. In Case 1, seven regular UWB anchors (in green) and three Google WiFi devices are used to show the performance of fusion with high-accuracy ranges. The numbers of UWB and WiFi devices were chosen in order to fully cover the scenario with at least three anchors or AP ranging data in the whole area. In Case 2, two UWB anchors in bad locations (in red wine colour) were added to the Case 1. In this scenario, UWB and WiFi will complement their information and improve the geometry of the problem by having the reference points more evenly distributed. Thus, with multi-technology fusion in trilateration the problem becomes over-determined for each point, and it also has more reference points distributed over a wider area (which means that the coverage of the overall system increases). As opposed to the scenario shown in Figure 3a, Figure 3b shows a realistic scenario with one regular UWB device, one WiFi AP and three LTE base stations distributed on the laboratories. With this deployment, the high-precision information availability is reduced to a small area. LTE is used in order to augment the availability of the positioning service. Nonetheless, with this deployment, a high precision can only be achieved when there are three high-precision reference points in range. When LTE is used, a low precision location is provided, which is still better than a full outage in location provision.

## 5. Results

In this section, the results obtained from each case are described separately in order to demonstrate the performance of multi-technology fusion. Moreover, the different searching methods of MLE are executed in order to observe some location characteristics for each Case.

### 5.1. Results from Multi-Technology Fusion

#### 5.1.1. Case 1: High-Density Deployment with Good UWB Conditions

**Figure 4:**
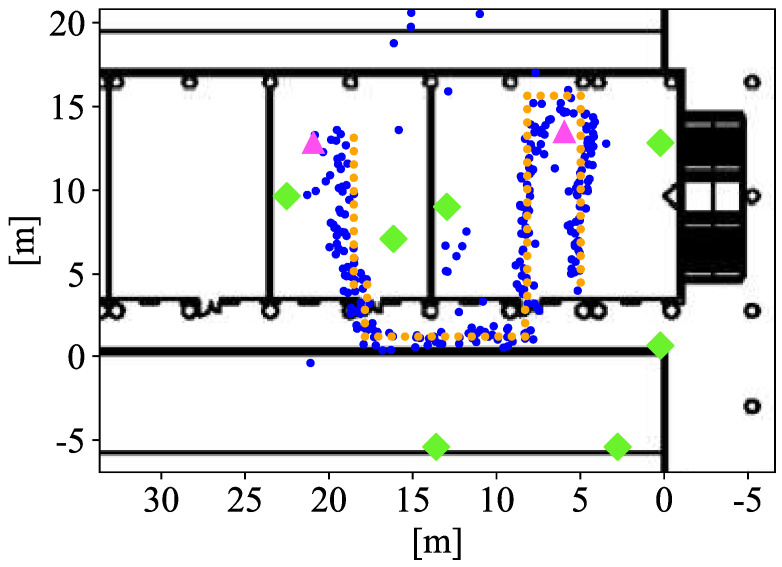
Localisation performance of Case 1 in 2D.

**Figure 5:**
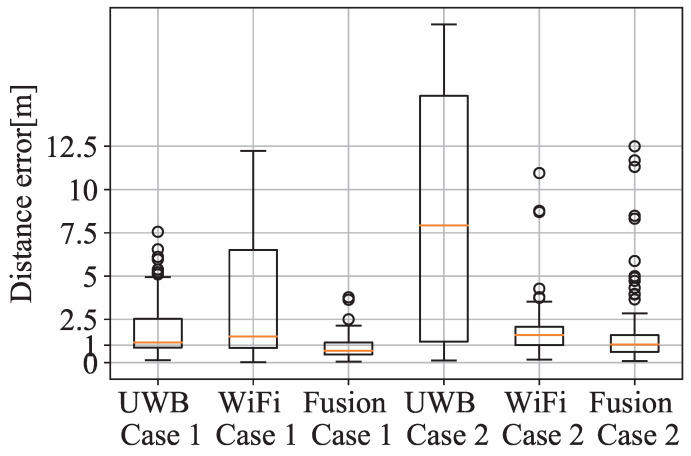
Horizontal error distribution of UWB + WiFi FTM for Cases 1 and 2.

In this first experiment, the set-up represents a scenario with a high-density of UWB reference points, such that at any point the visibility of at least four anchors is guaranteed. Moreover, all the anchors are in a location with good propagation conditions, favouring a low ranging error. Figure 3a represents the scenario, with the UWB anchors and the captured location data for one trajectory (yellow and orange dots). Figure 4 shows in 2D the performance of system with the estimated locations (blue dots) against the ground truth points (yellow dots). The UWB (green diamonds) and WiFi (pink triangles) illustrate where the devices are placed. Figure 5 shows the location accuracy achieved with UWB, WiFi FTM and the fusion of both in this case compared with the ground truth. In Figure 5, fusion median (yellow line), standard deviation (rectangle height), and outliers (circles) improves significantly from UWB and WiFi isolated cases. UWB localisation is better than WiFi in this case. Despite having UWB anchors in good conditions, the positioning error has an average near one meter. This meter-level accuracy is due to multipath effects that affect both UWB and WiFi FTM (which has a lower accuracy). When using fusion, the accuracy improves, with a lower average and much lower variance.

#### 5.1.2. Case 2: High-Density Deployment with 2 UWB in Bad Locations

As in the previous case, the set-up ensures the positioning service provided by UWB with the ranging information of four anchor most of the time. However, in this case, two of the anchors are installed in locations where a partial blocking of the anchors leads to bad propagation conditions due to NLoS. This situation can be common in the real world, where quick deployments are completed for situations such as emergencies or temporary events. The bad deployment causes these anchors to report ranges with a higher error. The data captured for this case are illustrated in Figure 3a as yellow dots. In this case, the error of the only-UWB location service is much larger than in the previous case as shown in Figure 5. Again, fusion median and standard deviation improve against isolated technology localisation.

**Figure 6:**
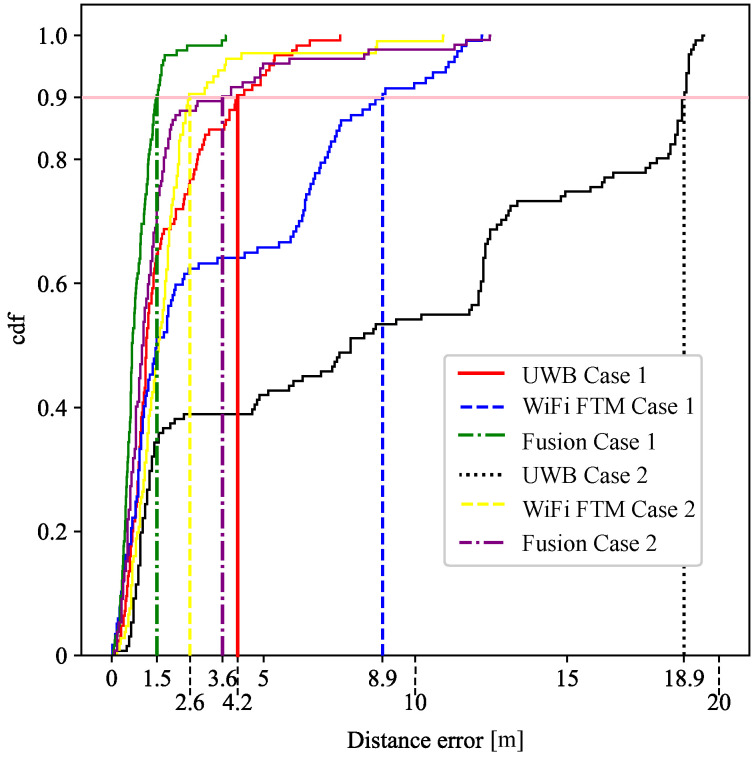
CDF of the horizontal error for Case 1 and 2.

A cumulative density function (CDF) of the error is given to illustrate and compare the performance of Cases 1 and 2 as shown in Figure 6. The pink horizontal line represents 90% sample line, and the error for using fusion in Case 1 drastically improves the horizontal error. However, the outliers introduced by UWB in Case 2 have worsened the fusion performance and WiFi, in this case, locates better the user in isolation.

#### 5.1.3. Case 3: Low-Density Deployment of High-Precision Technologies

**Figure 7:**
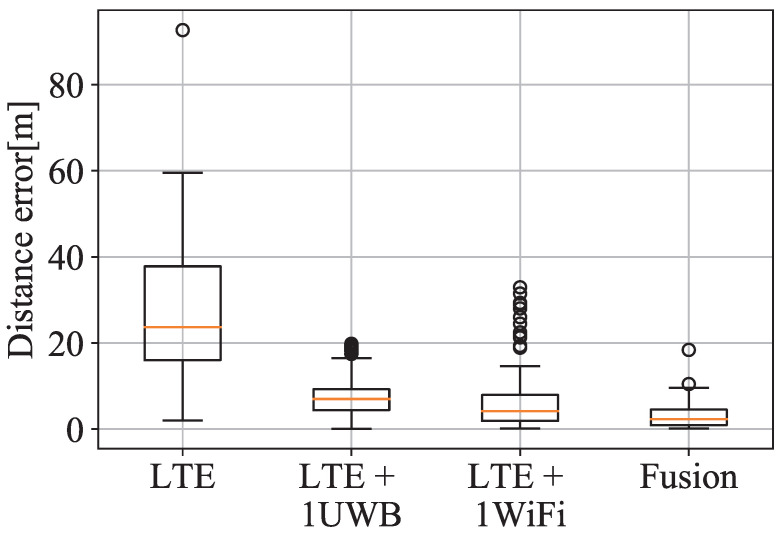
Horizontal error distribution of LTE and fusion for Case 3.

In this third case, the scenario is set up as a more realistic situation with less dense high-precision devices than in the other Cases. In this case, the low-density of devices in the scenario makes it impossible to locate a target by using the high-precision information from UWB and WiFi devices, since the visibility of at least four reference points is not guaranteed. Therefore, in this case, the missing ranges are complemented with LTE ranges, which are less accurate. Figure 3b represents, in light blue dots, the positions where the location service is provided by using the LTE data. In Figure 7, there is a comparison of the location error between the only LTE and multi-technology fusion between LTE with UWB anchors and WiFi AP ranging information.

**Figure 8:**
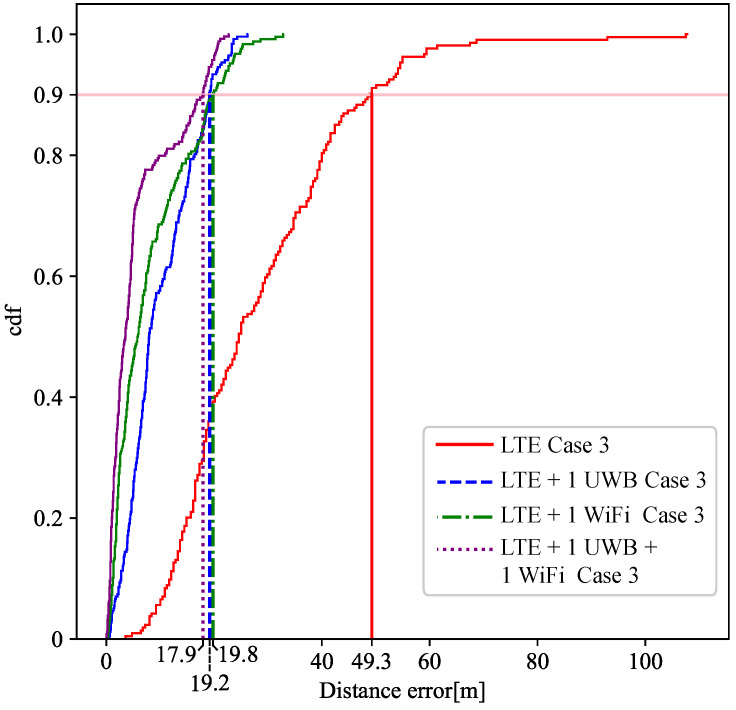
CDF of the horizontal error for Case 3.

In addition, the CDF of Case 3 has been also included when using fusion with 1 UWB anchor, 1 WiFi AP or LTE plus both technologies as shown in Figure 8. In this Case, precise ranging information greatly enhances the localisation accuracy of the multi-technology system.

**Figure 9:**
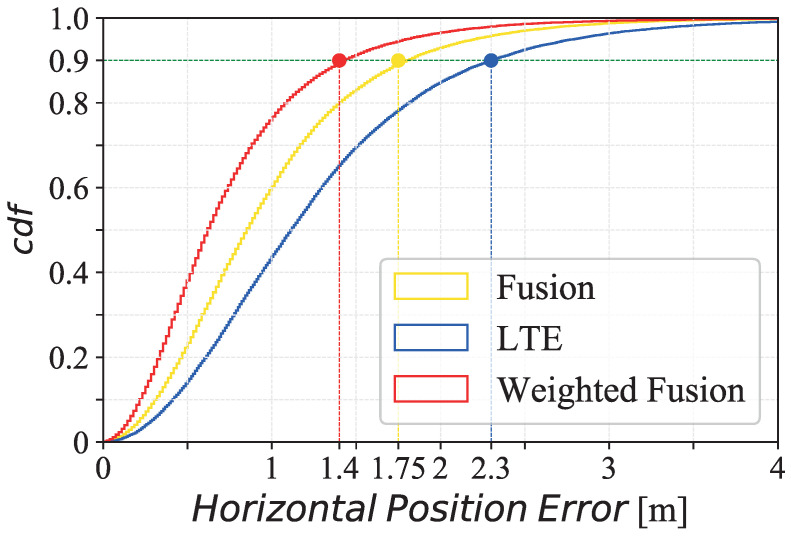
Horizontal Position Error of LTE (blue), fusion (yellow) and weighted fusion (red).

Comparing Figure 9 (imported from [10]) with Figure 8, the fusion algorithm stands out in both works showing the benefits of using high-precision data in areas only covered by low-precision ranging technology such as LTE. In Case 3, just as in the simulation of [10], LTE provides a worse precision performance when it is used in isolation. There is a contrast between the results obtained in this work and in [10] because in the simulation the case of the study was ideal in which some real-world conditions such as reflections, clutters, and interferences, were omitted.

### 5.2. Comparison of the MLE Searching Methods for Positioning

Searching methods may provide different solutions to weight the sources. Nelder–Mead, COBYLA, and SLSQP are linear methods which may perform better for estimating the standard deviation of the sources. In addition, L-BFGS-B is designed for large problems and TNC provides a rougher estimate to achieve a faster converge. Thus, L-BFGS-B and TNC may perform a priori worse than the rest. In this section, a comparison of the performance of the different searching methods for the MLE are shown in Table 3 in the three different cases. All the results are obtained by using multi-technology fusion in trilateration. In addition, the different searching methods are compared with a non-weighted LS solution to show the performance of the MLE in positioning problems. Table 3 shows the mean (μ), standard deviation (σ), the 80% of the cumulative error (CDF) in meters and the time elapsed for each iteration in milliseconds.

## 6. Discussion

In this section, the results are discussed showing the advantages of using multi-technology fusion. Moreover, the results obtained for the different searching methods are reviewed providing a guideline on which is the most convenient.

### 6.1. Performance of Multi-Technology Fusion

As seen in the previous section on Figure 5 and Figure 7, the error obtained by multi-technology fusion improves substantially with respect to single-technology positioning performance. With fusion, more measurements over-determine the WLS algorithm and also the geometry of the reference points enhances from a denser deployment. The over-determination of the localisation problem might not enhance the system performance with the inclusion of MLE if a RP appear intermittently, in which case MLE drastically reduces the impact of this intermittent RP.

On the other hand, the combination of isolated high-precision ranging technology with low-precision technology such as LTE shows a considerable improvement in all the possible aspects, such as mean, standard deviation, or the magnitude of the outliers, as seen in Figure 7. Despite LTE being a coarse precision ranging technology, it fills the lack of ranging information to solve the WLS algorithm for location augmenting the coverage area where location is provided. Therefore, taking advantage of the fact that several technologies are already deployed in the measurement scenario (and also in many real-world situations), the multi-technology fusion technique can be used to exploit different deployments, improving accuracy, coverage, and reducing the cost of new deployments.

### 6.2. MLE Search Methods

Regarding the MLE algorithm, it does not improve the target location performance overall when all the devices are in good visibility and propagation conditions (such as Case 1), instead, it slightly worsens the positioning error, as seen in Table 2. This is because MLE assigns a low initial score to the new sources. Nevertheless, the benefits of MLE appear in more realistic scenarios where input data introduces outliers (Cases 2 and 3). In Case 2, the use of MLE, with the methods of Nelder–Mead, COBYLA or SLSPQ, improve the location system performance in all the statistical metrics proposed in the Table 2 and SLSQP has a very similar time resolution without using MLE. In Case 3, it is noticeable that the MLE reduces the standard deviation error, although it increases the mean error. This is expected due to MLE reducing the impact of data with higher variances. Thus, despite reducing the impact of the standard deviation, the offsets introduced by multipath predominate on the location estimation. Again, the SLSQP search method is very similar to the non-weighted method. Hence, L-BSGS-B and TNC both show not to be suitable for positioning. In contrast, SLSQP search method proves better than the rest of the methods for improving the location performance against outliers or bad propagation conditions that are very typical for indoors scenarios.

The positive results obtained with MLE can be further improved using techniques, such as Kalman filters [24], or complementing the weight calculations with additional contextual information, such as the knowledge of LOS/NLOS conditions (obtained, for instance, with machine learning) [36].

## 7. Conclusions

In this work, the main objective is to present multi-technology fusion with MLE as a weighting algorithm in a real scenario. Thanks to the fusion technique, the presence of multiple technologies can be used to improve location in diverse ways: with a higher precision and with a higher availability. When the number of high-precision reference points is high, fusion provides an over-determination that allows a higher precision. In cases where the number of high-precision reference points is low (for instance, in the border of deployments, or in sparse deployments), multi-technology fusion allows using low-precision and highly available technologies, such as LTE, to complement the reference points and do trilateration to achieve a high availability on the localisation service.

Moreover, the proposed technique does not need any additional hardware apart from the receivers for each technology that will be present in most mobile devices in the near future. Thus, fusion allows to reduce costs in positioning infrastructure deployments due to a lower density requirement of high-precision devices.

To validate the weighting technique with MLE, tests with real deployments were completed in three different Cases. MLE is presented in this paper as a technique that reduced the impact of outliers for precise positioning. Only in ideal cases with very good condition deployments, the error slightly increases. SLSQP stands out as the best search method for MLE in positioning problems.

## Figures and Tables

**Table 1:** Overview of acronyms.

Acronym	Definition
5G	Fifth generation
AP	Access Points
AR	Augmented Reality
BLE	Bluetooth Low Energy
FCC	Federal Communication Commission
FTM	Fine-Time Measurement
GNSS	Global Navigation Satellite Systems
GPS	Global Satellite System
IoT	Internet of Things
L-BFGS	Limited-memory Broyden–Fletcher–Goldfarb–Shanno
LoS	Line of Sight
LTE	Long Term Evolution
NLoS	Non Line of Sight
MLE	Maximum Likelihood Estimator
PANS	Positioning And Networking Stack
RSSI	Received Signal Strength Indicator
RTT	Round-Trip Time
ToA	Time of Arrival
UE	User Equipment
UWB	Ultra-Wide Band
WLS	Weighted Least-Square

**Table 2:** Overview of indoor positioning technology.

Technology	Access Point	Positioning Accuracy	Positioning Method	Advantages	Disadvantages
Cellular network	Cellular tower	>30 m	Trilateration	World-wide coverage; No extra infrastructure needed	Low-precision > 100 m
UWB	UWB anchor	cm-m	Trilateration	Robust against multpath; high-accuracy; easy-deployment	High-cost
WiFi-FTM	Router	cm-m	Trilateration	Low cost; high-accuracy	Not yet widely deployed
Bluetooth	Beacon	m	Trilateration; fingerprinting	Low cost; easy-deployment	Low-stability
INS	N/A	m	PDR	Self-sufficient	Accumulative error; Smartphone-based calculation
Geomagnetism	N/A	m	Fingerprinting	No infrastructure; low-cost; ubiquitous	Need data collection; affected by temporal electrical equipment;

**Table 3:** Comparison of the search methods in both scenario.

		Nelder-Mead	L-BFGS-B	TNC	COBYLA	SLSQP	No Weighting
Case 1	μ [m]	1.14	1.46	1.43	1.14	1.14	1.07
	σ [m]	0.77	1.2	0.99	0.77	0.77	0.67
	80% cdf error [m]	1.63	1.84	1.93	1.63	1.63	1.45
	Time elapsed [s]	0.103	0.065	0.169	0.200	0.067	0.070
Case 2	μ [m]	0.98	1.52	1.52	0.96	0.95	1.11
	σ [m]	0.67	1.74	1.74	0.67	0.67	0.84
	80% cdf error [m]	1.3	1.83	1.83	1.25	1.23	1.46
	Time elapsed [s]	0.125	0.079	0.176	0.225	0.082	0.078
Case 3	μ [m]	18.7	19.08	18.36	18.7	18.7	18.14
	σ [m]	9.65	10.75	10.14	9.66	9.66	10.71
	80% cdf error [m]	27.84	27.36	26.88	27.84	27.84	25.71
	Time elapsed [s]	0.109	0.059	0.194	0.254	0.052	0.050

## Data Availability

Not applicable.
